# The Risk of Massage

**Published:** 1883-11

**Authors:** 


THE RISK OF "MASSAGE." DR. JULIUS ALTHAUS, M. D., Senior Physician to the Hospital for Epilepsy and Paralysis, Regent's Park, deprecates the abuse of massage, a practice often now employed where it can be of no service. "It is well known that at various times epilepsy, idiocy, and some forms of insanity, have been treated by massage and gymnastics; but fortunate, ly we now hear very little of such therapeutical aberrations.

It appears to me that diseases of the brain and spinal cord, must, on account of the anatomical situation of these organs, be inaccessible to the influence of massage, which can only be applicable to more superficial parts of the body. Apart from this, however, it is important to consider that many of the most important diseases of these organs are of an inflammatory or irritant character, either primarily or secondarily; and this should make it self-evident that massage should not be used for their treatment, even if the suffering parts could be reached by it. I will here only allude to many forms of cerebral paralysis from hæmorrhage, embolism, and thombosis, which are followed by sclerosing myelitis of the pyramidal strands; and most forms of primary, lateral, posterior or insular sclerosis of the spinal cord.

That which may be good for developing and strengthening healthy muscles or muscles which have been enfeebled by disuse or certain local morbid conditions, etc., is not for that reason suitable for the treatment of muscular paralysis owing to central disease. In most cases of lateral and insular sclerosis, which are, unfortunately, now much treated with massage and exercises, rest is indicated rather than active exertion; and overstraining of the enfeebled muscles acts prejudically on the state of the nervous centers. I have recently seen quite a number of instances in which the central disease had been rendered palpably worse by procedures of this kind; and, in a case of cerebral paralysis, which was sometime ago under my care, the patient had, after four such sittings, been seized with collapse, which nearly carried him off."—*British Medical Journal.*

